# Persistence Pays Off: Live Birth after Uterus Transplant, Overcoming Recurrent Pregnancy Loss with Cerclage Placement

**DOI:** 10.3390/jcm12206463

**Published:** 2023-10-11

**Authors:** Liza Johannesson, Sophie Anderson, J. Michael Putman, Robert T. Gunby, Lilly Zhang, Giuliano Testa, Anthony R. Gregg

**Affiliations:** 1 Baylor Annette C. and Harold C. Simmons Transplant Institute, Baylor University Medical Center, Dallas, TX 75246, USA; 2 Department of Obstetrics and Gynecology, Baylor University Medical Center, Dallas, TX 75246, USA; 3 Fordham University, New York, NY 10458, USA; 4 Fertility Center of Dallas, Baylor University Medical Center, Dallas, TX 75246, USA; drjmputman@aol.com (J.M.P.);; 5 Department of Obstetrics and Gynecology, Prisma Health Midlands, Columbia, SC 29201, USA

**Keywords:** cerclage, cervical insufficiency, graft rejection, inflammation, uterus transplantation, venous outflow

## Abstract

Recipients of uterus transplantation have unique factors that may increase their risk of cervical insufficiency. This report describes a uterus transplant recipient with cervical insufficiency resulting in two second-trimester miscarriages. After McDonald cerclages (one that failed), she underwent an interval transabdominal cerclage and delivered a healthy term child in her third pregnancy. The longitudinal information of this case provides observations from which we can propose testable hypotheses that address venous outflow and inflammation. This case also suggests that there could be a role for prophylactic cerclage placement at the time of transplantation.

## 1. Introduction

Uterus transplantation (UTx) is the only treatment for women with absolute uterine-factor infertility who desire to experience pregnancy and childbirth. Since the first live birth in the US following UTx in 2017 [1], more than 30 babies have been born in the US [2,3].

Although pregnancies after UTx generally lead to a live birth, several specific risk factors have been suggested in this population [4,5,6]. One of these risk factors is cervical insufficiency [7,8]. In the general obstetric population, the incidence of cervical insufficiency is estimated to be <1% [9], and in women with a history of prior mid-trimester miscarriages, 8% [10]. Interventions for cervical insufficiency include cervical length surveillance, medical therapies, surgical intervention, or a combination of these [11,12]. The possible surgical interventions include transvaginal [13] or transabdominal [14,15] cervical cerclage [16]. The indications for a cerclage include past obstetric history (mid-trimester loss or preterm birth attributed to cervical insufficiency), ultrasound examination (short cervix with or without funneling), or physical examination (rescue or emergency cerclage) [17,18,19]. Multiple metaanalyses [20,21] and studies [22,23] have shown the success of a cervical cerclage placement, depending on the indication of the cerclage [24]. There are no reports of cerclage in UTx recipients. 

UTx recipients have unique factors that may put them at a higher risk of cervical insufficiency compared to women with a native uterus. The denervation of the uterus at transplantation and exposure to warm and cold ischemia are exclusive to the transplanted uterus. Furthermore, the transplanted uterus largely lacks ligamentous support due to the absence of supportive tissues, such as the cardinal ligaments, and possesses a unique blood supply [25]. The cervical tissue is potentially exposed to inflammation associated with organ rejection, cervical biopsies, and healing. In addition, the recipient’s microbiome that surrounds the cervix likely differs from that of the donor. These characteristics potentially impede the normal progressive remodeling of the cervix and thus contribute to cervical insufficiency. On the other hand, due to donor selection, other known clinical risk factors for cerclage, such as prior preterm birth and prior cervical procedure (e.g., loop excision or conization), are absent. 

In this report, we present a case of a UTx recipient with cervical insufficiency resulting in two second-trimester miscarriages prior to delivering a healthy term child. She was treated for cervical insufficiency with two McDonald cerclages [26] and a transabdominal cerclage procedure. Her third pregnancy was successful. Cervical insufficiency and its surgical management have never been described in detail in a UTx patient. 

## 2. Case Report

### 2.1. Recipient and Donor Background

In 2018, a 28-year-old woman with Mayer–Rokitansky–Küster–Hauser syndrome underwent UTx as one of 20 cases in the Dallas Uterus Transplant Study (NCT02656550). At the time of her surgery, 11 previous cases had been performed at the center. The recipient was diagnosed with Mayer–Rokitansky–Küster–Hauser at the age of 16. Prior to UTx, she underwent self-dilation to create a functional neovagina. No surgical vaginal interventions had been performed, and at the time of UTx, the recipient’s vagina measured 6 cm. The nonrelated directed living donor was 33 years old at the time of surgery. She was a gravida 2, para 2. Both conceptions were unassisted and she had no history of abnormal uterine anatomy. She had two previous caesarian deliveries; the first cesarean delivery was in gestational week 39 due to nonreassuring fetal heart tones remote from delivery and placental abruption; the second cesarean delivery was planned in gestational week 38 due to the previous cesarean section. The donor had no history of cervical insufficiency.

### 2.2. Uterus Transplantation Surgery

The techniques for uterus donation and transplantation have previously been described in detail [27]. In this specific case, the uterine graft was retrieved with bilateral vascular pedicles of the uterine arteries with segments of the anterior portion of the internal iliac arteries, and inferior uterine veins with corresponding portions of the internal iliac veins. The graft vessels were anastomosed to the bilateral external iliac vessels of the recipient. No anastomoses of the superior uterine veins were performed [25]. During the transplantation, the uterine graft was exposed to a total ischemic time of 8 h and 13 min (cold ischemic time, 6 h and 56 min, and warm ischemic time, 1 h and 17 min). The patient received induction therapy with Thymoglobulin (4.5 mg/kg in three divided doses) and 1000 mg methylprednisolone and was maintained on tacrolimus (with a goal trough level of 5 to 8 ng/mL for the first 3 months and 3 to 5 ng/mL thereafter) and azathioprine. She received prophylactic enoxaparin sodium for 6 weeks after UTx followed by low-dose aspirin (81 mg) daily throughout pregnancy and the postpartum period. In addition, she received a 3-month course of valganciclovir 450 mg daily for cytomegalovirus prophylaxis and trimethoprim–sulfamethoxazole (80/400 mg daily) against *Pneumocystis jirovecii* pneumonia. No postoperative complications occurred, and the recipient was discharged from the hospital on postoperative day 6. The recipient started regular menses 4 weeks post-surgery. 

### 2.3. Monitoring for Acute Cellular Rejection and Treatment of Rejection Episodes

To detect possible graft rejection, cervical biopsies were routinely taken from the recipient with a Tischler biopsy punch (MedGyn, Addison, IL, USA). Biopsies were performed on postoperative day 5, weekly through week 4, and once monthly before and after pregnancy. According to our study protocol, biopsies were planned to occur twice during pregnancy (at 12–14 weeks and at 24–26 weeks of gestation). During the first two pregnancies, the patient was biopsied only at 12–14 weeks, and during the third pregnancy, her cervix was biopsied as planned. Biopsies were graded as negative, borderline, mild, moderate, or severe [28].

**Figure 1:**
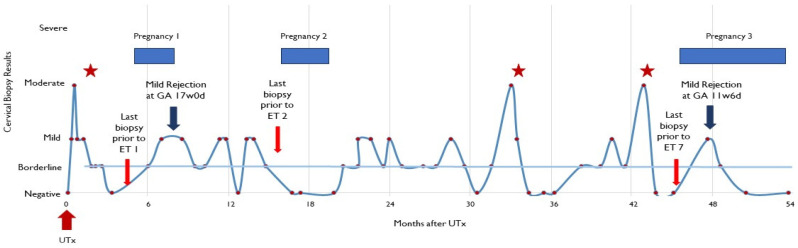
Detailed overview of cervical biopsies and results following uterus transplantation. ET—embryo transfer; UTx—uterus transplant; GA—gestational age. Stars represent rejection episode treated with intravenous methylprednisolone 500–1000 mg on day 1 and 500 mg on days 2 and 3. Dots represents cervical biopsies.

The recipient had three episodes of moderate rejection, which occurred at 3 weeks, 33 months, and 43 months after UTx (Figure 1). No episode of moderate rejection occurred during ongoing pregnancy. The episodes were treated with intravenous methylprednisolone 500–1000 mg on day 1 and 500 mg on days 2 and 3. Repeat cervical biopsies were performed 1 week after rejection treatment until the rejection episodes were resolved. The recipient also received treatment (an increase in daily dose of azathioprine, from 50 to 75 mg, and the addition of daily 5 mg prednisone) for a mild rejection episode in the first trimester of her third pregnancy. This patient had cervical biopsies on 52 separate occasions. On 19 of these dates (36.5%), the biopsies showed either mild or moderate rejection.

### 2.4. Vaginal Stricture

The recipient was diagnosed with the common complication of a vaginal stricture following UTx [7]. The stricture was first noted 2 weeks after UTx and later noted 59, 92, and 115 days after UTx. The patient started self-dilation 4 weeks post UTx. Self-dilation failed to resolve the vaginal stricture. At each cervical biopsy procedure, mechanical dilation was performed with ring forceps to gain access to the cervix. In addition, she underwent surgical intervention at 2 weeks and 2 months post UTx and at the time of McDonald placement for the first and second pregnancies. 

### 2.5. In vitro Fertilization and Embryo Transfer

In vitro fertilization (IVF) of the recipient was performed four times during the period prior to and following transplantation. Our IVF protocols included ovarian stimulation by agonist antagonist conversion with estrogen priming. Ovarian stimulation was performed with the use of exogenous gonadotropins–recombinant follicle-stimulating hormone (Gonal-f, EMD Serono, Rockland, MA, USA) and human menopausal gonadotrophin (Menopur, Ferring Pharmaceuticals, Parsippany, NJ, USA). The insemination of mature oocytes was performed with ICSI. The first IVF procedure was performed 2 months prior to UTx. Sixteen oocytes were retrieved, twelve of which matured, and four euploid day-5 blastocysts were stored. The second and third IVF procedures occurred 30 and 35 months following UTx (before the third pregnancy). All embryos underwent preimplantation genetic testing, and we ultimately collected six euploid embryos. During embryo transfer, a single-warmed, good-quality, expanded blastocyst was transferred under ultrasound guidance while the patient was awake. Transfer was performed using the Guardia Pro ET catheter system (Cook Medical, Bloomington, IN, USA). All embryos were placed into the uterine cavity. When embryo implantation occurred, luteal support was given throughout the first trimester.

**Figure 2:**
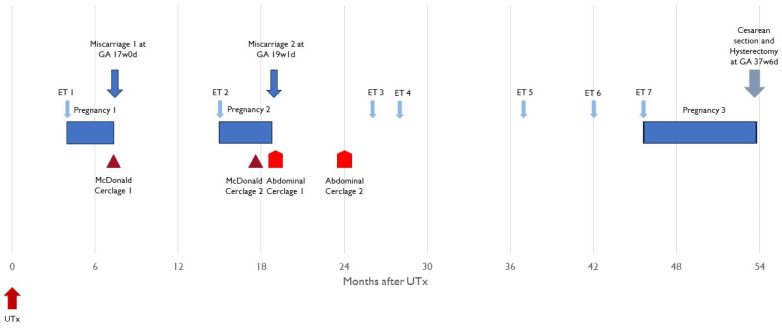
Overview of embryo transfers, pregnancies, and timing of cerclage interventions. ET—embryo transfer; UTx—uterus transplant; GA—gestational age.

The first embryo transfer occurred 3.9 months after UTx and ultimately resulted in a second-trimester miscarriage (Table 1). A second embryo was transferred 15.4 months after UTx, which also resulted in a second-trimester miscarriage. Four subsequent embryo transfers (including one with a low level mosaicism) either failed to result in pregnancy or resulted in a biochemical pregnancy. The seventh embryo transfer, 3 years and 10 months after UTx, resulted in a live birth. All embryo transfers and their results are displayed chronologically in Table 1 and Figure 2.

### 2.6. Overview: First Pregnancy

Clinical pregnancy was determined 4 weeks after the patient’s first embryo transfer (4.8 months after UTx). At 16 weeks and 5 days gestation, the patient underwent a routine transvaginal sonographic assessment to determine the cervix length. The recipient was found to have a shortened cervix (<5 mm), and an ultrasound-indicated McDonald cerclage was placed (Figure 1). In the operating room, the amniotic membrane was reduced, and the recipient received prophylactic antibiotics. Two days later, ultrasound examination determined fetal demise. The patient was without symptoms and the cerclage was found intact. An antiphospholipid antibody panel was performed, revealing elevated phosphatidylserine IgM (34 GPL U/mL) and IgG (15 GPL U/mL). After vaginal delivery, the fetus was sent to pathology. No evidence of abnormalities was found, and development was deemed appropriate for gestational age. The etiology of this demise remains elusive. 

### 2.7. Overview: Second Pregnancy

**Figure 3:**
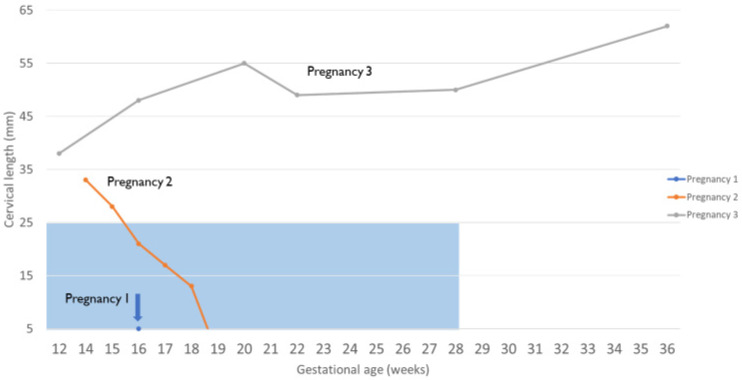
Changes in cervical length during pregnancy as measured using vaginal ultrasonography. The blue transparent box symbolizes a short cervix (<25 mm before 28 weeks of pregnancy).

Seven months after the first miscarriage, the patient underwent a second embryo transfer, resulting in her second pregnancy (15.4 months after UTx). The decision was made to place a history-indicated prophylactic McDonald cerclage during gestational week 14. At this time in gestation, she was also noted to have a partial placenta previa. At 15 w 6 d gestation, the partial placenta previa was confirmed, and cervical beaking was identified. At 16 w 6 d, it was determined that the partial placenta previa had been resolved. However, the cervical beaking persisted and a short cervix was noted at 21 mm (Figure 3). At 19 w 1 d, the cervix was <5 mm, and the patient underwent an exam under anesthesia. The membranes were found to be visible. When the previous cerclage suture was removed, a laceration of the cervix could be seen, which extended to the fornix on the right side. It was decided that an exam-indicated McDonald cerclage revision would be futile. Therefore, an exam-indicated open abdominal cerclage was attempted. Within 24 h of the cerclage placement, fetal demise was diagnosed. The abdominal cerclage was found to be intact. The patient was reopened, and the suture material was removed. Pathology revealed fetal membranes with necrotizing chorioamnionitis. 

### 2.8. Open History-Indicated Cerclage

Five months after the second fetal demise, an interval abdominal cerclage was placed (24.8 months after UTx). The abdomen was opened through a subumbilical midline incision (following the previous incision at UTx). The bladder was mobilized, and the bilateral uterine arteries identified. A suture (Mersilene 5 mm tape) was inserted with an atraumatic needle between the vascular pedicle, containing the uterine arteries and veins, the uterus, through to the peritoneum on the posterior surface of the uterus, and the medial to the vascular pedicle on the contralateral side. The suture was tied anteriorly.

### 2.9. Overview: Third Pregnancy

Embryo transfer during postoperative month 45 resulted in a successful and uneventful term pregnancy (45.8 months after UTx). There were no signs of cervical shortening, placenta previa, or cervical funneling (Figure 3). The patient successfully underwent a planned cesarean section and hysterectomy at 37 weeks and 6 days. Of note, the hysterectomy was performed as the patient wanted no further pregnancy. At delivery, the baby girl weighed 2880 g and measured 48 cm in length. The APGAR score was 8 and 9 at 1 and 5 min, respectively, and no surgical intervention or neonatal intensive care unit stay was necessary. After 2 days, both the mother and child were discharged from the hospital. The placenta weighed 450 g (50th percentile for gestational age). Histology revealed villous maturity appropriate for gestational age, <5% focal infarct, two-vessel umbilical cord, negative for funisitis, membranes, negative for chorioamnionitis, and disrupted maternal surface consistent with the diagnosis of placenta accreta. 

## 3. Discussion

In our research series of the first 14 UTx recipients achieving live birth, we had two patients with cervical insufficiency (14%) [7,29]. Our first experience with cervical insufficiency in this population resulted in a live birth after an examination (prolapsing membranes)-indicated McDonald cerclage. This first case has been previously described and offered no special insights into this patient population [7]. In this report, we focused our attention on the second of our patients with cervical insufficiency, as the longitudinal information of this particular case provides interesting observations from which we can propose testable hypotheses. 

Since the initiation of UTx, there have been concerns of whether the transplanted uterus would respond similarly to a native uterus after embryo implantation. Live birth rates of around 70% following UTx in larger studies are reassuring and suggest that the transplanted uterus is capable of functioning at least as well as a native uterus [2]. Due to their impact on the transplanted uterus, factors such as ischemia-reperfusion injury during procurement, altered uterine blood supply and venous outflow, systemic immunosuppression, and graft inflammation continue to fuel concerns surrounding UTx. 

Thus far, our observations within the UTx recipient population have not uncovered evidence to support the notion that the procedure itself, with its innate ischemia-reperfusion injury, impacts cervical competence or pregnancy outcomes [2,4,7]. Importantly, we observed that venous outflow was unique in our two patients with cervical insufficiency. Both had no superior uterine vein anastomoses and relied exclusively on the inferior uterine vein bilaterally for venous outflow. More work is needed, and likely a collaborative effort will be required to better understand whether superior uterine venous outflow is important in maintaining cervical competence. 

Our case report offers insights into a possible role for inflammation in the second trimester. Of the three established pregnancies, we suggest focusing on the first and third. The course of the second pregnancy is difficult to interpret due to the long cervical laceration, which likely resulted in cervical shortening in the second trimester and made the McDonald cerclage ineffective. Pregnancies 1 and 3 were preceded by histologic evidence of moderate rejection at about 14 and 12 weeks prior to embryo transfer, respectively. Both of these episodes resolved with intravenous corticosteroids. Importantly, the biopsy preceding each successful embryo transfer was negative for any sign of rejection, which demonstrated rejection treatment efficacy. After the established pregnancies, surveillance for rejection continued. In the first pregnancy, an episode of mild rejection occurred at 2.5 (14 weeks gestation) weeks prior to the ultra sound identification of the cervical insufficiency (at 16 weeks and 5 days gestation). This episode was treated with an increase in maintenance immunosuppression (tacrolimus). During the third pregnancy, mild rejection was confirmed at 12 weeks of gestation. This episode was treated with an increase in existing maintenance immunosuppression (tacrolimus) and the addition of oral corticosteroids. Subsequent biopsy 4 weeks later demonstrated the resolution of the rejection episode with a borderline result.

Cervical biopsy is the accepted method for monitoring rejection in uterus transplant recipients. There are currently no biomarkers or functional measures of uterine graft rejection. Although patients undergo biopsies and post-biopsy focal inflammation is assumed, this has no clinical implications for success. In our prior work, despite undergoing biopsies according to our established protocols, most patients deliver without cervical insufficiency. At this time, our combined experience in the research protocol and clinical service has yielded only two patients with cervical insufficiency 2/25 uterus transplants. 

Our data should be interpreted optimistically because cerclage in our third pregnancy seemed capable of overcoming inflammation-induced cervical insufficiency, unusual venous outflow, or both. Our very first cerclage patient went on to a successful pregnancy with delivery at 30 w 6 d gestation [7]. The patient we present here lost her first pregnancy 2 days after cerclage but was not in labor and showed no evidence of chorioamnionitis or cerclage failure (e.g., bleeding or cerclage pulling through, dilation, or effacement). The cause of this demise remains elusive to us. The abdominal cerclage after the second pregnancy and before the third pregnancy was necessary due to the laceration, and we believe the laceration was the primary factor in the second pregnancy loss. The open abdominal cerclage placed between pregnancy 2 and 3 was a success when measured using the ultrasound assessment of cervical length. This pregnancy was successful despite the presence of an ongoing mild inflammatory response and resulted in delivery of a healthy newborn at 37 w 6 d. 

A major strength of our case is the suggestion of a possible mechanism for ultrasound-identified second-trimester cervical shortening and/or dilation in patients with either a transplanted or a native uterus. If inflammation is a concern, one wonders whether medical prophylaxis with drugs other than those used to prevent rejection can be undertaken to reduce cervical inflammation in those with a native uterus. Furthermore, one wonders whether there is a role for cervical biopsy in those patients suspected of having cervical insufficiency as part of their surveillance strategy. Our case introduces a unique testable *null* hypothesis in the UTx population: venous outflow at the time of UTx is not important in the etiology of cervical insufficiency. The primary limitation of our study was the realization that in order to address this hypothesis, a larger patient population is required. We hope other groups can collaborate and that this hypothesis can be definitively addressed. Finally, UTx is a limited-time transplant, and the goal is a short interval between the transplant to pregnancy. This case suggests there could be a role for prophylactic cerclage placement at the time of transplantation. Looking retrospectively, our patient may have had a shorter interval between the transplant and successful pregnancy with a prophylactic cerclage at transplantation. More work is needed to address our observations and determine surveillance strategies and prophylactic interventions. 

## Figures and Tables

**Table 1:** Embryo transfers and pregnancy outcome.

Embryo Transfer	Months after UTx	Embryo Implantation	Pregnancy Outcome	Comment
1	3.9	Yes	Miscarriage at GA 17 w 0 d	Ultrasound- and exam-indicated McDonald cerclage at GA 16 w 5 d
				Fetal demise at GA 17 w 0 d
2	15.4	Yes	Miscarriage at GA 19 w 1 d	History-indicated McDonald cerclage at GA 14 w.
				Abdominal rescue cerclage at GA 19 w 1 d
				Fetal demise at GA 19 w 1 d
	24.8			History-indicated open abdominal cerclage placed
3	26.0	No		
4	28.0	No		
5	37.3	Yes	Biochemical pregnancy	
6	42.0	Yes	Biochemical pregnancy	
7	45.7	Yes	Live birth at GA 37 w 6 d	Pregnancy success

Abbreviations: GA—gestational age; w—weeks; d—days.

## Data Availability

Data are contained within the article.

## Abbreviations

The following abbreviations are used in this manuscript:

IVF	in vitro fertilization
UTx	uterus transplantation
